# Assessing the severity of conjunctivochalasis in a senile population: a community-based epidemiology study in Shanghai, China

**DOI:** 10.1186/1471-2458-11-198

**Published:** 2011-03-31

**Authors:** Xingru Zhang, Qingsong Li, Haidong Zou, Jinjuan Peng, Caicai Shi, Huanming Zhou, Guili Zhang, Minhong Xiang, Yijie Li

**Affiliations:** 1 Department of Ophthalmology, Putuo Hospital, Affiliated Shanghai Traditional Medicine University, Shanghai 200062, China; 2 Department of Ophthalmology, Shanghai First People's Hospital, Affiliated Shanghai Jiaotong University, Shanghai 200080, China

## Abstract

**Background:**

In a previous hospital-based study, conjunctivochalasis was diagnosed in 85.24% of 1416 patients aged 1 to 94 years and in 98.5% of patients aged 60 or older. This report is the first to present data from a large scale epidemiologic study on conjunctivochalasis in a community-based population, thereby helping to better identify the severity of conjunctivochalasis in the general population.

**Methods:**

This community-based epidemiologic study was conducted to evaluate prevalence rates and related factors of conjunctivochalasis among people over 60 years old in the CaoYangXinCun community, Shanghai, China. Cluster sampling was used in randomly selected local residents aged no less than 60 years. A cross-sectional study using a slit-lamp ophthalmoscope for examination was carried out from September 2008 to October 2008. A modified grading system based on the well-accepted Meller and Tseng's system was used. SPSS10.0 software was used for data and statistical analysis.

**Results:**

A total of 2110 residents took part in this study, with a response rate of 94.85%. Among these, 930 cases were confirmed as conjunctivochalasis, with a prevalence rate of 44.08%. The prevalence rate increased with age (X^2 ^= 10.44, P < 0.01). A total of 1762 eyes were confirmed as conjunctivochalasis eyes. Of these eyes, 943 were classified as Grade I (53.52%), 647 as Grade II (36.72%), 162 as Grade III (9.19%), and the remaining 10 eyes as Grade IV (0.57%). The conjunctiva usually accumulated on the nasal and temporal areas of the conjunctival sac (944 eyes, 53.58%).

**Conclusions:**

Although the rate estimated in the present study is not as high as reported in the former hospital-based study, conjunctivochalasis is considered a common age-related eye disease, deserving more attention to its early diagnosis.

## Background

The original term conjunctivochalasis was coined by Hughes in 1942, describing a redundant, loose, nonedematous inferior bulbar conjunctiva interposed between the globe and the lower eyelid[1]. In 1986, Liu emphasized frequent tearing (epiphora) as a symptom of conjunctivochalsis after a thorough study in 15 patients. She proposed two main mechanisms of tearing, and suggested the new definition as "An isolated bilateral condition in which redundant conjunctival tissue overlies the lower eyelid margin or covers the lower punctum, and it causes tearing by mechanically disrupting the normal flow of tears."[2]. In 1995, the relationship between conjunctivochalasis and keratoconjunctivitis sicca (KCS, generally known as Dry Eye Syndrome) was further explored by Höh and associates[3]. They graded conjunctivochalasis according to lid-parallel conjunctival folds (LIPCOF); the risk of contracting KCS was identified as increasing with the grade of LIPCOF. However, Meller and Tseng thought that the KCS defined by Höh might merely be an unstable tear film, and that the LIPCOF classification overlooked the fact that the location of the redundant conjunctiva on the lower lid varies, and the size of the conjunctival fold can be increased by downgaze or digital compression onto the globe[4]. Therefore, they proposed a grading system for conjunctivochalasis in their review[4], that has subsequently been used by Di Pascuale, Yokoi and associates[5,6].

In 2009, Mimura and associates [7] described a hospital-based study using a modified Meller and Tseng's grading system: Grade 0 (no persistent fold); Grade 1 (a single, small fold); Grade 2 (two or more folds but not higher than the tear meniscus); Grade 3 (multiple folds and higher than the tear meniscus). Grading was carried out separately for the temporal, middle and nasal areas of the conjunctiva. In addition, the extent of conjunctivochalasis was further classified with punctual occlusion, and conjunctival fold changes in downgaze and digital pressure[7]. In Mimura's report, Grade 1 to Grade 3 conjunctivochalasis was diagnosed in 85.24% of 1416 consecutive patients aged 1 to 94 years attending their outpatient clinic. The proportion reached a level as high as 98.5% in patients aged no less than 60 years. Therefore, it is inferred that conjunctivochalasis is a quite common public health concern. However, several Chinese ophthalmic doctors, including us, share the similar clinical experience that less than 50% of old patients that come to our clinics are diagnosed with conjunctivochalasis, and less than 50% of the identified patients need further medical treatment. Until now, no report has presented large scale epidemiologic data for conjunctivochalasis in community-based populations, data that may help to more precisely evaluate the severity of conjunctivochalasis in the general population.

In the present study, we describe results of an epidemiologic investigation carried out in the older Chinese residents of the CaoYangXinCun community, Shanghai, from September 1st to October 31st, 2008, using a modified grading system for conjunctivochalasis based on Meller and Tseng's system. The main results of this manuscript were originally published in Chinese language as following: [Epidemiologic study of conjunctivochalasis in populations equal or over 60 years old in Caoyangxincun community of Shanghai, China, [8]] Zhonghua Yan Ke Za Zhi. 2009, 45:793-8. [Article in Chinese]

## Methods

### Study population

The CaoYangXinCun community is located in the western part of Shanghai city. A population census, conducted at the end of 2007, showed that a stable urban population of 88,973 was living in this community. Among them, 22,128 were older residents not less than 60 years old, accounting for 24.87% of the total population. The average annual income of these residents was at the medium level of Shanghai residents.

A cluster sampling method was used for this investigation. Five subordinate groups, or committees, from this community were randomly selected for sampling. A total of 2225 residents, not less than 60 years old, were included in these 5 committees as study candidates.

### Field investigation

The field investigation was carried out near each committee from September 1st to October 31st, 2008. The study team included one lead ophthalmic doctor who had prior experience organizing large scale epidemiologic studies (HZ), two trained ophthalmic doctors as the investigators, two assistant ophthalmic doctors, and three assistant physicians. Volunteers from the local committee government helped to inform each participant by oral and written notice.

The field investigations ran from 8:00 AM to 4:00 PM on each study day. The assistant physicians collected general information from study candidates, including name, gender, birth date, race, educational level, etc. The assistant ophthalmic doctors then documented eye symptoms, and obtained best corrected visual acuity of both eyes using the Snellen chart. Finally, the investigators examined patients' eyelids, conjunctiva, cornea, lacrimal lake, anterior chamber, pupil, and lens using slit-lamp biomicroscopy, and assessed the grade of conjunctivochalasis in each eye. All information obtained was documented in a self-made questionnaire.

The study was carried out with the approval of the Institutional Review Board of Putuo Hospital Affiliated Shanghai Traditional Medicine University, and was performed in accordance with the Declaration of Helsinki of 1975 and its 1983 revision. Every resident provided written informed consent for inclusion in the study.

### Grading system and classification criteria

The modified grading system for conjunctivochalasis used in this study was previously introduced by XZ in a Chinese journal[9], with detailed information shown here in Table 1. The location of conjunctivochalasis is specified as T, M and N similar to Meller and Tseng's system if conjunctivochalasis was found in the temporal, the middle (or inferior to the limbus), and the nasal aspect of the low lid, respectively. Grade criteria F were set as the basic grading criteria, irregardless of the conjunctival fold occurring in any of the 3 locations. The height of the tear meniscus was measured by calibration on the slit lamp. The normal value was set as no less than 0.3 mm. If either of the two eyes was diagnosed with conjunctivochalasis, the patient was recorded as a conjunctivochalasis patient.

**Table 1:** Modified Meller and Tseng's grading system for conjunctivochalasis used in the present study*

	Basic grading criteria		Supplement grading criteria		
		
	Folds versus Tear Meniscus Height(F)	Symptoms(S)	Punctual Occlusion and Tear Meniscus Height(O)	Height/extent of chalasis Changes in Downgaze(G)	BUT(B)
0	No persistent fold	No	Without occlusion	No difference	>= 10s
1	Single, small fold	No	Without occlusion and tear meniscus height <= 0.3 mm	No difference	>= 10s
2	Two or more folds and not higher than the tear meniscus	Mild	Nasal location with partial occlusion and irregular tear meniscus	mildly increases in downgaze	6-9s
3	Multiple folds and higher than the tear meniscus	Medium	Nasal location with complete occlusion and discontinuous tear meniscus	Significant increase in downgaze	4-5s
4	Multiple folds, higher than the tear meniscus, and causing exposure problems	Severe	Nasal location with complete occlusion and no tear meniscus	Severely increase in downgaze	<= 3s

*Symptoms indicate dryness, foreign body sensation, and epiphora, evaluated by the patients themselves BUT:break-up time(seconds)

This grading system for conjunctivochalasis obeys the following rule: if the clinical appearance of a certain patients corresponds to F2 + S2, or F2+ any two of O2, G2 and B2, the patient will be diagnosed as Grade II conjunctivochalasis, and so forth with Grade0, Grade I, Grade III, or Grade IV.

Grade II, Grade III, and Grade IV are also defined as "clinical significant conjunctivochalasis".

### Statistics

Before the field investigation, the leader of the team trained the two investigators in examination methods and diagnosis and grading criteria using typical conjunctivochalasis eye photographs. After training, the two investigators checked and recorded the diagnosis of conjunctivochalasis in 40 eyes of 20 randomly selected people, respectively. A high consistency between their diagnoses (к = 0.93) was found.

The general characteristics, signs and symptoms of conjunctivochalasis patients and non-conjunctivochalasis patients were compared using the Pearson line × list chi square test or tendency chi square test. The tests were considered to be statistically significant at p < 0.05 (SPSS v10, Chicago, IL). The Snellen fractions were converted to a log scale (LogMAR) for statistical analysis using the method of Holladay and Prager[10]. The t-test was utilized to compare the logMAR eyesight of conjunctivochalasis eyes and non-conjunctivochalasis eyes.

### Results

A total of 2,110 residents, not less than 60 years old, participated and completed the study, with an inclusion rate of 94.85%. Of these, 835 were male and 1,275 were female. Average age of these 2110 participants was 72.05 ± 7.88 years.

A total of 930 participants were confirmed as conjunctivochalasis patients, and the prevalence rate of conjunctivochalasis in these older residents was 44.08%. The prevalence of clinically significant conjunctivochalasis was found in 374 participants, and the prevalence rate was 17.73%. The average age of the conjunctivochalasis group was 72.58 ± 7.53 years, significantly higher than that of the remaining non-conjunctivochalasis patients (71.61 ± 8.14 years, t = 2.83, P < 0.01). The average age of the male gender group in conjunctivochalasis patients was 72.57 ± 7.89 years, almost the same as the female group 72.63 ± 7.20 years (t = 48.68, P = 0.08). The highest prevalence rate of conjunctivochalasis was found in the eldest group, (Chi-square for trend value = 41.10, P < 0.01), as shown in Table 2.

**Table 2:** The prevalence rate of conjunctivochalasis in 4220 eyes of 2110 residents

Age	total number of eyes	number of eyes that suffered from conjunctivochalasis	prevalence rate (%)
60-70	1493	561	37.25
71-80	1984	881	44.41
81-90	701	299	42.65
91-	42	21	50.00

A total of 1762 eyes were confirmed as conjunctivochalasis eyes. Of these eyes, 943 were classified as Grade I (53.52%), 647 as Grade II (36.72%), 162 as Grade III (9.19%), and the remaining 10 eyes as Grade IV (0.57%). The percentages of different grades in different age groups are shown in Table 3. The average logMAR visual acuity of the 1762 conjunctivochalasis eyes was 0.42 ± 0.43, not statistically lower than that of the remaining 2458 non-conjunctivochalasis eyes (0.42 ± 0.45, t = 0.18, P = 0.86).

**Table 3:** The number and percentage of different grading in different age groups of 1,762 conjunctivochalasis eyes

Age	Total No.(%)	Grade I		Grade II		Grade III		Grade IV	
		
		**No**.	%	**No**.	%	**No**.	%	**No**.	%
60-65	294(100)	183	62.24	89	30.27	19	6.46	3	1.02
66-70	267(100)	148	55.43	97	36.33	21	7.87	1	0.37
71-75	379(100)	223	58.84	121	31.93	34	8.97	1	0.26
76-80	502(100)	245	48.80	204	40.64	50	9.96	3	0.60
81-85	222(100)	104	46.85	91	40.99	25	11.30	2	0.90
86-90	77(100)	30	38.96	38	49.35	9	11.70	0	0.00
91-95	17(100)	9	52.94	5	29.41	3	17.60	0	0.00
≥96	4(100)	1	25.00	2	50.00	1	25.00	0	0.00
Total	1762(100)	943	53.52	647	36.72	162	9.19	10	0.57

The location of conjunctivochalasis was most frequently found as nasal + temporal in the 1,762 conjunctivochalasis eyes. In the 10 Grade IV conjunctivochalasis eyes, more severe redundant conjunctiva was found, as 50% of them were of the temporal+middle+nasal location type, as shown in Table 4. In 267conjunctivochalasis eyes with redundant conjunctiva in the middle location, 30.33% of these (81 eyes) showed visual acuity (Snellen chart) lower than 0.3. This percentage is significantly higher than in the remaining 1495 conjunctivochalasis eyes (24.01%, 1495 eyes X^2 ^= 4.83, P = 0.02.)

**Table 4:** The number and percentage of positions of piled conjunctiva in various Grades of 1,762 conjunctivochalasis eyes

Position of piled conjunctiva	Total		Grade I		Grade II		Grade III		Grade IV	
	
	**No**.	%	**No**.	%	**No**.	%	**No**.	%	**No**.	%
1	944	53.58	520	29.51	338	19.18	84	4.77	2	0.11
2	391	22.19	207	11.75	157	8.91	24	1.36	3	0.17
3	194	11.01	107	6.07	39	2.21	43	2.44	5	0.28
4	160	9.08	67	3.80	90	5.11	3	0.17	0	0.00
5	34	1.93	21	1.19	6	0.34	7	0.40	0	0.00
6	29	1.65	13	0.74	16	0.91	0	0.00	0	0.00
7	10	0.57	8	0.45	1	0.06	1	0.06	0	0.00
Total	1762	100	943	53.52	647	36.72	162	9.19	10	0.57

*: position type of piled conjunctiva in conjunctival sac: 1. nasal + temporal part. 2. only temporal part, 3. temporal +middle+ nasal part, 4. only nasal part, 5. temporal +middle part, 6. middle+ nasal part, 7. only middle part

## Discussion

Efforts are ongoing to explore the novel mechanisms of conjunctivochalasis in experimental and clinical studies, as well as to clarify grading systems[11-14]. The controversy over Meller and Tseng's grading system focused primarily on its neglect of patients' complaints or dry eye symptoms, which are considered to be closely associated with conjunctivochalasis[4-7,15-17]. Grade 1 in their system indicates a single, small fold, which often occurs as a normal aging variation, and its clinical significance was not clear. Grade 2 indicates two or more folds and not higher than the tear meniscus, which also occurs in normal persons more than 70 years of age. Therefore, we doubt that some asymptomatic, normal older people would be graded as conjunctivochalasis patients according to this system, resulting in the high proportion (98.5%) in the Mimura and associates' study[7]. As conjunctivochalasis progresses, punctual occlusion and unstable tear film inevitably occur[2,7], leading to epiphora, feeling of dryness and foreign body sensation. For this reason, we chose these 3 symptoms as a supplemental grading criteria (S) into our modified grading system. For the same reason, the well-accepted diagnostic criteria of unstable tear film-BUT is set as another supplemental grading criteria (B) of conjunctivochalasis. The other 2 supplemental grading criteria are almost the same as Meller and Tseng's system: punctual occlusion and tear meniscus height (O) and height/extent of chalasis changes in downgaze (G). Patients diagnosed as Grade II or more according to this modified system suffer from mild to severe symptoms and/or abnormal tear appearance, and regular follow-up, pharmacotherapy or surgery will be suggested to them. Therefore, Grade II, Grade III and Grade IV stages are defined as "clinically significant conjunctivochalasis". Grade 3 stage in Meller and Tseng's system is divided into Grade III and Grade IV in this modified system. Grade IV conjunctivochalasis corresponds to the most severe condition, where surgery is always mandatory.

The prevalence rates of total conjunctivochalasis and clinically significant conjunctivochalasis in the present study were 44.08% and 17.73%, respectively. The rates are much lower than those reported in the previous Japanese study[7], but align with what we have observed in our clinical practice. However, after these results were reported in a Chinese-language journal, some Chinese ophthalmic doctors were of the opinion that the prevalence rates were still higher than they expected. Reasons may include the following: (1) Grade I and Grade II conjunctivochalasis, with none or only mild symptoms, accounted for 90.24% of total patients (older Chinese patients are always reluctant to find medical help for these slight discomforts); (2) primary symptoms of conjunctivochalasis are similar to dry eye disease. If the doctor neglects signs such as piled conjunctiva or an abnormal lacrimal river, misdiagnosis is inevitable. It should be emphasized that common treatment methods for dry eye disease do not apply to severe conjunctivochalasis, which can only be treated with specialized surgery[4,18-20]. We hope the prevalence rate found in this epidemiologic study will have a positive impact on ophthalmic doctors and the diagnosis of conjunctivochalasis.

We confirmed that conjunctivochalasis is an age-related disease that became more extensive with age (Table 2 and Table 3), as the previous study concluded[7]. Although conjunctivochalasis patients always complained of blurred vision, the average far-sighted visual acuity in their eyes was not statistically lower than that of non-conjunctivochalasis eyes. Reasons may be related to an abnormal lacrimal river and unstable tear film. However, when one co-relates the visual acuity with the position of the redundant conjunctiva, those eyes with middle location conjunctivochalasis were more likely to have worse visual acuity. Murube proposed the reason that middle location disease interferes with the lower tear meniscus more severely[21]. Generally, the conjunctival sac near the internal and external canthus is much more shallow than the middle part, so redundant conjunctiva tends to pile up at the nasal and temporal areas, always obstructing the inferior puncta, and is followed by abnormal tear excretion[22]. When punctal obstruction occurs, the symptoms of conjunctivochalasis appear relatively earlier[23]. In Grade IV conjunctivochalasis eyes, almost all the inferior conjunctiva is piling, leading to severe symptoms as shown in Table 4.

The possible shortcomings of the present study should not be neglected. Although the prevalence rates of conjunctivochalasis using the modified grading system seem to be in accord with our clinical experience, the validity of this novel system should be further evaluated in future studies. We believe that our grading system is a useful attempt to link different clinical appearances of conjunctivochalasis with corresponding medical treatments. We also believe that future investigations with other objectives and more reliable grading systems that include all possible elements for defining the severity of conjunctivochalasis will evolve, as confirmed by Meller and Tseng[14].

## Conclusions

From this epidemiologic study, we estimated the prevalence rates of conjunctivochalasis as 44.08% in a senile Chinese population, using a modified grading system based on the well-accepted Meller and Tseng system. Although the rate is not as high as reported in a former hospital-based study, conjunctivochalasis is confirmed as a common eye disease in the senile population. The clinically significant conjunctivochalasis accounts for 17.73% of all patients, indicating that more attention may be warranted for the early diagnosis of this condition.

## List of abbreviations

KCS: keratoconjunctivitis sicca; LIPCOF: LIPCOFlid-parallel conjunctival folds; BUT: break-up time

## Competing interests

The authors declare that they have no competing interests.

## Authors' contributions

XZ participated in the design of the grading system and this epidemiologic study, contributed to the discussion and edited the manuscript. QL participated in the design of this epidemiologic study, collected research data. HZ (Haidong Zou) participated in the design of the study, collected research data, contributed to the discussion and wrote the manuscript. JG, CS, HZ (Huanming Zhou), GZ, MX, and YL participated in the research data collection. All authors have read and approved the final manuscript.

## Pre-publication history

The pre-publication history for this paper can be accessed here:

http://www.biomedcentral.com/1471-2458/11/198/prepub
